# A Comb-Drive Actuator Driven by Capacitively-Coupled-Power

**DOI:** 10.3390/s120810881

**Published:** 2012-08-07

**Authors:** Chao-Min Chang, Shao-Yu Wang, Rongshun Chen, J. Andrew Yeh, Max T. Hou

**Affiliations:** 1 Institute of NanoEngineering and MicroSystems, National Tsing Hua University, 101, Sec. 2, Kuang-Fu Rd., Hsinchu 30013, Taiwan; E-Mails: chaomin.chang@gmail.com (C.-M.C.); rchen@pme.nthu.edu.tw (R.C.); jayeh@mx.nthu.edu.tw (J.A.Y.); 2 Department of Mechanical Engineering, National United University, 1, Lienda, Miaoli 36003, Taiwan; E-Mail: shaoyuwang.nuu@gmail.com

**Keywords:** actuation mechanism, capacitive coupling, electrostatic actuators

## Abstract

This paper presents a new actuation mechanism to drive comb-drive actuators. An asymmetric configuration of the finger overlap was used to generate capacitive coupling for the actuation mechanism. When the driving voltages were applied on the stators, a voltage would be induced at the rotor due to the capacitive coupling. Then, an electrostatic force would be exerted onto the rotor due to the voltage differences between the stators and the rotor. The actuator's static displacement and resonant frequency were theoretically analyzed. To verify the design, a comb-drive actuator with different initial finger overlaps, *i.e.*, 159.3 μm and 48.9 μm on each side, was fabricated and tested. The results show that the actuator worked well using the proposed actuation mechanism. A static displacement of 41.7 μm and a resonant frequency of 577 Hz were achieved. Using the actuation mechanism, no electrical connection is required between the rotor and the outside power supply. This makes some comb-drive actuators containing heterogeneous structures easy to design and actuate.

## 1. Introduction

Electrostatic comb-drive actuators, which feature easy design, fabrication and implementation, are an important type of actuators. They have been used in various application fields, such as optical communication [1], biomedical engineering [2], wireless communication [3] and nanotechnology [4]. A longer traveling distance and a larger force output are two major concerns in developing comb-drive actuators. In the last two plus decades, many research efforts were devoted to improve these two major performance factors. These efforts can be grouped into four main directions: optimizing the finger shape [5], modifying the spring shape [6], modifying the configuration of finger overlaps [7] and creating new actuation methods [8].

Recently, besides the abovementioned directions, some low stiffness materials, such as polydimethylsiloxane (PDMS) and SU-8, were used to construct the springs of comb-drive actuators to obtain larger displacements [9,10]. Although the expected advantage of using low stiffness materials (usually nonconductive) is achieved, the comb electrodes of the rotor are inevitably isolated. An extra process of metal deposition is required to make the rotor's finger electrodes electrically connected with outside circuits once again. During operations, the thin metal layers likely have the risk of delamination. The drawback of the bilayer springs makes the heterogeneous comb-drive actuators unreliable. A suitable method should be developed to cancel the drawback.

**Figure 1.**
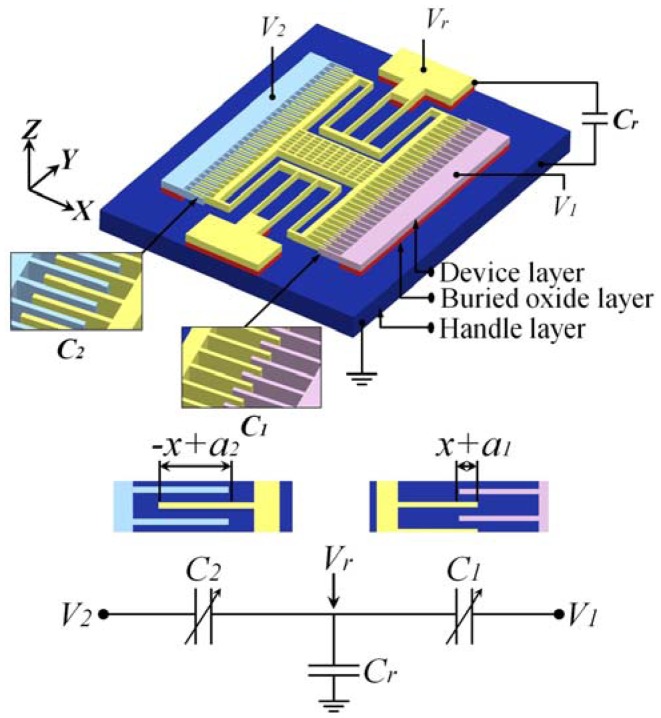
Schematics of the comb-drive actuator with the actuation mechanism of capacitively-coupling-power supply. Different initial overlaps, *a*_1_ and *a*_2_, form different initial capacitances, *C*_1_ and *C*_2_. With the capacitance, *C_r_*, which is formed between the rotor and the handle layer, a capacitive circuit is observed. Applying voltages, *V*_1_ and *V*_2_, onto the stators and grounding the handle layer, a voltage, *V_r_*, will be induced at the rotor. As long as *V*_1_ and *V*_2_ are not the same, the rotor will be moved by the net electrostatic force generated by the comb electrodes.

To eliminate the drawback, a capacitively-coupled-power driven comb-drive actuator (see Figure 1), whose rotor requires no electrical interconnection, was proposed in this article. In the following sections, the concept and design of the actuator will be explained and analyzed first. Then, the tests for the fabricated actuators will be described, and the results will be discussed. Finally, the conclusions will be made.

## 2. Capacitively-Coupled-Power Supply

One way to deliver power to the rotor without electrical interconnection is using a capacitive coupling with the stators. Capacitively-coupled-power has been successfully used to drive microrobots in the MEMS area [11]. To achieve this, we treated the stators and rotor of the comb-drive actuator as a sequence of insulated electrodes, as shown in Figure 1. When different voltages are applied to the stators, the rotor, the stators and the handle layer form a capacitive circuit. The potential (*V*_r_) induced on the rotor is determined by the voltages applied on the two stators using the following equation:

(1) $$V_{r}=\frac{V_{1}{\text{C}}_{1}+V_{2}C_{2}}{C_{1}+C_{2}+C_{r}}$$

where *V*_1_ and *V*_2_ represent the applied voltages, and *C*_1_, *C*_2_ and *C_r_* represent the capacitances. Although *V_r_* is floating, its value can be definitely determined by Equation (1), *i.e.*, it is dependent on *V*_1_ and *V*_2_. Possible variation of *V_r_* may arise from the parasitic capacitance that exists in the device. This issue can be removed by careful design to avoid any parasitic capacitance.

If the voltages *V*_1_ and *V*_2_ are different, the voltage differences between the rotor and the low-voltage and high-voltage stators will be different. The voltage differences bring about different attraction forces on two sides of the rotor. Then, the rotor will be moved to the position where the balance between the mechanical and the electrostatic forces achieves.

By Equation (1), the influence of *C_r_* can be observed. As *C_r_* approaches zero, then:

(2) $$V_{r}=\frac{V_{1}{\text{C}}_{1}+V_{2}C_{2}}{C_{1}+C_{2}}$$

and *V_r_* will be determined by *V*_1_, *V*_2_, *C*_1_ and *C*_2_. This can be achieved by removing the handle layer under the movable part and isolating the rotor's anchors from the stators' anchors. Extra etching and bonding processes are required to do so. In this case, the asymmetric configuration of finger overlap is necessary to generate an initial difference between *C*_1_ and *C*_2_, and then an initial difference between |*V_r_* – *V_1_*| and |*V_r_* – *V_2_*|. Without the asymmetric configuration, the actuator will not be actuated. As *C_r_* approaches infinity, then:

(3) $$V_{r}≅\frac{V_{1}{\text{C}}_{1}+V_{2}C_{2}}{C_{r}}$$

Namely, *V_r_* will approach zero. This can be achieved by significantly increasing the areas of the rotor's anchors. It is easy to estimate *V_r_* in this case. However, the excessive device area makes this case not suitable to be implemented. In this article, *C_r_* was neither close to zero nor infinity, hence Equation (1) was used as the basic formula to obtain *V_r_*.

Note that when non-conductive materials are used to construct the springs of comb-drive actuators, *C_r_* is determined by the area of the movable structure. *C_r_* will never approach infinity. Equation (1) will also be used as the basic formula to obtain *V_r_*.

## 3. Design and Analysis

### 3.1. Design

An asymmetric comb-drive actuator with different initial engagements, *a*_1_ and *a*_2_, between rotor and stator fingers on each side was designed to achieve different initial *C*_1_ and *C*_2_, as shown in Figure 1. Such an asymmetric configuration guarantees the actuation as *C_r_* = 0, which can be achieved by improving the device design and the fabrication process, as mentioned above.

### 3.2. Influence of Surrounding Electrostatic Field

**Figure 2.**
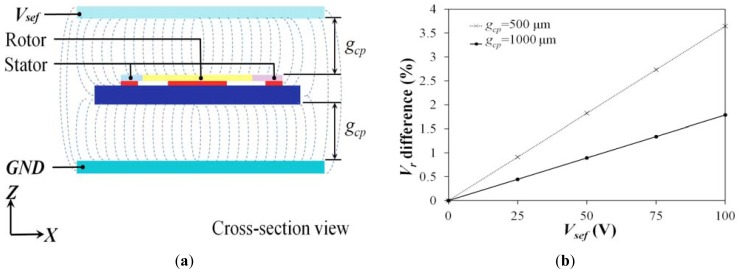
(**a**) The testing model: a proposed actuator is sandwiched between a pair of parallel plates. A voltage of *V_sef_* is applied on the upper plate, and the lower plate is grounded. A surrounding electrostatic field will be created as a possible outside interference. (**b**) The *V_r_* difference induced by the surrounding electrostatic field.

In this paper, we assume the surrounding electrostatic field is comparatively small, thus its influence was omitted. To ensure the validity of the assumption, the influence of surrounding electrostatic field was evaluated by simulation analysis using CoventorWare software. Figure 2(a) shows the model on which we performed the evaluation. A comb-drive actuator using the proposed actuation mechanism was sandwiched between two imaginary parallel plates. A dc voltage was applied onto the parallel plates to create a surrounding electrostatic field. Two factors, *i.e.*, the distance between the actuator and the upper or lower plate, *g_cp_*, and the applied voltage, *V_sef_*, were taken into consideration. Figure 2(b) shows the simulation results. The value of *g_cp_* and *V_sef_* refer to actual conditions of IC packaging and operation. Typically, *g_cp_* is larger than 500 μm and *V_sef_* is less than 25 volts. In this case, the actuator is surrounded by an electrostatic field of 50 kV/m, and then about 0.9% *V_r_* difference will be induced. Hence, the influence of outside electrostatic field can be ignored.

### 3.3. Static Displacement

To simplify modeling, the electrostatic field between the movable and fixed fingers is approximated by the parallel plate model between the engaged parts of the comb fingers. Due to the configuration of the comb-drive, the capacitances between movable and fixed fingers in the high-voltage (*V*_1_) and low-voltage (*V*_2_) sides, as shown in Figure 1, can be derived as:

(4) $$C_{1}=\frac{2n{ɛ}_{\text{air}}h\left(x+a_{1}\right)}{g}$$

and:

(5) C2=2nɛhair(−x+a2)g

where *x* is the displacement of the rotor, *n* is the numbers of finger pairs, *ε_air_* is the permittivity of air, *h* is the finger height, *g* is the spacing between movable and fixed comb fingers.

The capacitance between the rotor and the handle layer can be written as:

(6) $$C_{r}=\frac{{ɛ}_{\text{air}}A_{\text{sus}}}{d}+\frac{{ɛ}_{ox}A_{\text{anch}}}{d}$$

where *A_sus_* and *A_anch_* are the areas of the rotor's suspended part and anchor, respectively. *ε_ox_* is the permittivity of silicon dioxide. *d* is the thickness of the silicon dioxide layer. Note that *C_r_* is a constant because of the constant *A_sus_* and *A_anch_*.

Assuming the rotor is a good conductor and the actuator is operated to obtain a static displacement, in the capacitive circuit shown in Figure 1, the total potential *U* existing in *C*_1_, *C*_2_ and *C*_r_ can be expressed as:

(7) $$U=\frac{1}{2}\left[C_{1}{\left(V_{1}-V_{r}\right)}^{2}+C_{2}{\left(V_{2}-V_{r}\right)}^{2}+C_{r}{V_{r}}^{2}\right]$$

The longitudinal force induced by the electrostatic potential is:

(8) $$F_{ex}=\frac{\partial U}{\partial x}$$

The restoring force of the folded spring [12] can be expressed as:

(9) $$F_{sx}=k_{x}\cdot x=\frac{2Ehb^{3}}{L^{3}}\cdot x$$

where *k_x_*, *E*, *b* and *L* represent the spring constant (longitudinal direction), Young's modules, width and length, respectively.

In equilibrium, the electrostatic force is balanced by the restoring force of the spring, *i.e.*, *F_ex_* = *F_sx_*. After some manipulation, the static displacement can be calculated as:

(10) $$x=\frac{{BT}_{1}}{{\left(a_{1}+a_{2}+C_{G}\right)}^{2}k_{x}-{BT}_{2}}$$

where

B = nε_air_h/g,
T_1_ = 2(a_1_S_1_ + a_2_S_2_ + C_G_S_3_)V_Δ_ + S_1_^2^ – S_2_^2^,
T_2_ = a_1_V_Δ_^2^ + 2S_1_V_Δ_ + a_2_V_Δ_^2^ – 2S_2_V_Δ_,
C_G_ = C_r_/(2B),
V_Δ_ = V_2_ – V_1_,
S_1_ = −V_Δ_a_2_ + V_1_C_G_,
S_2_ = V_Δ_a_1_ + V_2_C_G_, and
S_3_ = –V_1_a_1_ – V_2_a_2_.

From the above derivation, it can be found that unlike conventional comb-drive actuators, the resulting electrostatic force depends not only on the voltage applied, but also on the displacement of the comb. This will limit the travel range (just like it does for parallel plate capacitor).

### 3.4. Natural Frequency

The natural frequency of the asymmetric comb-drive actuator, like typical ones, is a function of its material properties, dimensions and boundary conditions. For simplicity, the actuator can be treated as a lumped system [12]. Then, its natural frequency can be expressed as:

(11) $$f=\frac{1}{2\pi }\sqrt{\frac{k_{x}}{m_{eq}}}$$

where *m_eq_* represents the equivalent mass of the actuator and equals $m_{\text{shuttle}}+\frac{1}{2}m_{\text{truss}}+\frac{96}{35}m_{\text{beam}}$. Here, *m_shuttle_*, *m_truss_* and *m_beam_* represent the masses of the shuttle, the single truss and the single beam, respectively.

## 4. Experiment and Results

**Figure 3.**
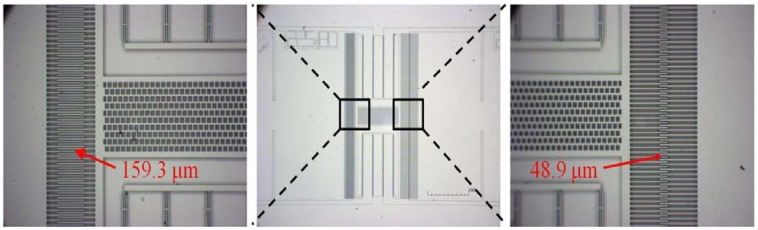
The fabricated comb-drive actuator with different initial overlaps. Here, *n* = 138, *h* = 50 μm, *L* = 1250 μm, *b* = 6 μm, *g* = 5 μm, *a*_1_ = 48.9 μm, *a*_2_ = 159.3 μm, *A_sus_* = 1.218 × 10^−6^ m^2^ and *A_anch_* = 7.414 × 10^−6^ m^2^.

To verify the new actuation mechanism, we fabricated some comb-drive actuators using the one mask process based on the silicon-on-insulator (SOI) wafer with a device layer of 50 μm and a buried oxide layer of 4 μm. The structure of the comb drive actuator was patterned and etched by deep reactive-ion etching (DRIE), then released with hydrofluoric acid (HF) etching to remove the sacrificial oxide. Figure 3 shows a microscope image of the actuator with asymmetric initial overlaps.

**Figure 4.**
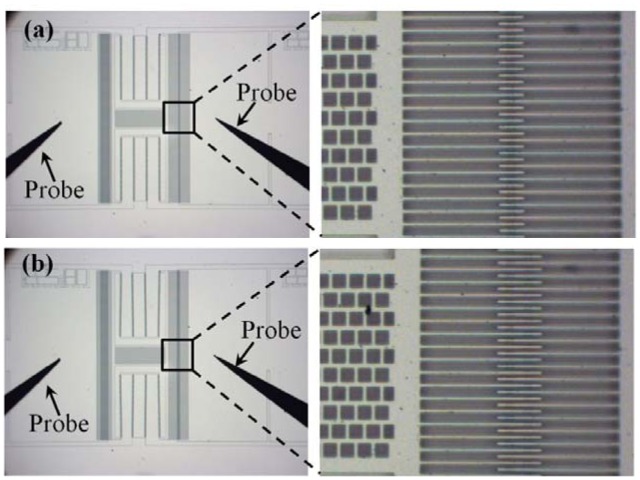
The actuation of the proposed comb-drive actuator. (**a**) Before and (**b**) after voltages were applied onto the stators, the rotor was static and moved a distance.

The feasibility of the actuation mechanism was demonstrated by the operation of the comb-drive actuator. The voltages were only applied on the stators and the rotor was completely insulated. The handle layer was grounded to ensure that its voltage is zero. Before applying the dc voltages, the rotor was static, as shown in Figure 4(a). Increasing the dc voltage at only one stator, *i.e.*, increasing *V*_1_, resulted in an increasing *F_ex_*, and thus the rotor motion (see Figure 4(b)).

**Figure 5.**
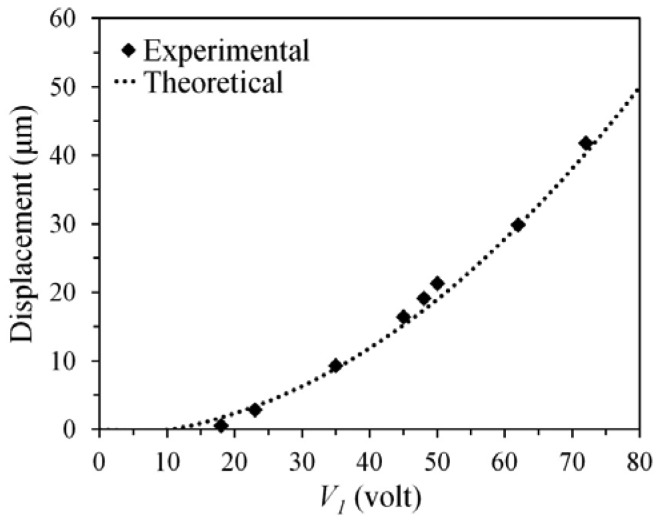
The estimated and measured displacements of the rotor. In this example, *V_2_* was fixed at 10 volt, *V_1_* was increased (from zero) to 72 volts.

Figure 5 shows the relationship between the displacement of the rotor and the applied voltage. The displacement of the rotor was measured using a high resolution microscope (on the probe station). The experimental results agree well with the estimated ones. When the applied voltage (*V_1_*) was increased to 72 volts, the displacement approached to 41.7 μm. The static responses verify that without electrical interconnections the rotor still can be driven using the capacitively-coupled-power delivery mechanism.

**Figure 6.**
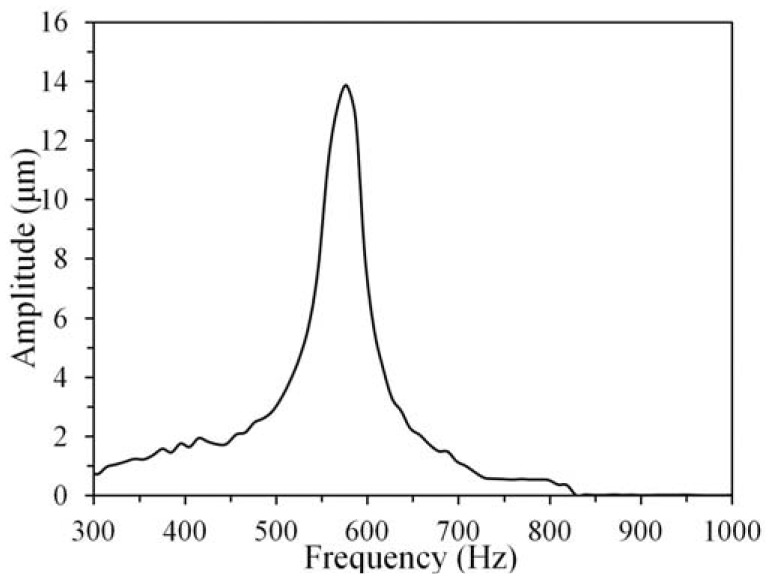
The measured dynamic response of the rotor.

The performance of the proposed actuation mechanism was also characterized by the dynamic response of the fabricated actuator. Through the capacitive coupling, the actuator was driven by a dc voltage (*V*_2_ = 5 volts) and an ac voltage (*V*_1_ = 20sin*ω*t volt, where *ω* represents the radian frequency) in air. The dynamic behavior was observed using a MEMS motion analyzer, *i.e.*, an in-plane strobe scope module. Figure 6 shows that the measured resonant frequency of the actuator—which was driven by the applied ac voltage with a frequency ranging from 300 to 1,000 Hz—is 577 Hz, which is close to the estimated value: 615 Hz. The dynamic response further demonstrates that the new actuation mechanism is feasible in dynamic actuation. Note that the difference between the theoretical and experimental results may result from the nonlinear relationship between the electrostatic force and the displacement *x*. However, the potential effects, such as spring hardening and softening, are out of scope of the current work and left to be a topic of future work.

## 5. Conclusions

Asymmetric comb-drive actuators were successfully designed, fabricated and implemented using the actuation mechanism, which takes advantage of the capacitive coupling inherent existing in the comb-drive actuator. The capacitively-coupled-power delivery was successfully used to drive the new actuators, whose static displacement and natural frequency can be predicted from the analytical solutions. The experimental results verified the theoretical analysis. Using this method, the rotor can be fully insulated, *i.e.*, the comb-drive actuators containing heterogeneous structures (e.g., flexible and insulating folded beams) become more practical and promising to have an impact on MEMS technology.
